# Random forest analysis of factors affecting urban carbon emissions in cities within the Yangtze River Economic Belt

**DOI:** 10.1371/journal.pone.0252337

**Published:** 2021-06-04

**Authors:** Zhaohan Wang, Zijie Zhao, Chengxin Wang

**Affiliations:** 1 College of Geography and Environment, Shandong Normal University, Jinan, Shandong, China; 2 School of Earth Sciences, University of Melbourne, Parkville, Melbourne, Victoria, Australia; 3 Australian Research Council Centre of Excellence for Climate Extremes, Melbourne, Victoria, Australia; Institute of Geographic Sciences and Natural Resources Research (IGSNRR), Chinese Academy of Sciences (CAS), CHINA

## Abstract

China became the country with the largest global carbon emissions in 2007. Cities are regional population and economic centers and are the main sources of carbon emissions. However, factors influencing carbon emissions from cities can vary with geographic location and the development history of the cities, rendering it difficult to explicitly quantify the influence of individual factors on carbon emissions. In this study, random forest (RF) machine learning algorithms were applied to analyze the relationships between factors and carbon emissions in cities using real-world data from Chinese cities. Seventy-three cities in three urban agglomerations within the Yangtze River Economic Belt were evaluated with respect to urban carbon emissions using data from regional energy balance tables for the years 2000, 2007, 2012, and 2017. The RF algorithm was then used to select 16 prototypical cities based on 10 influencing factors that affect urban carbon emissions while considering five primary factors: population, industry, technology levels, consumption, and openness to the outside world. Subsequently, 18 consecutive years of data from 2000 to 2017 were used to construct RFs to investigate the temporal predictability of carbon emission variation in the 16 cities based on regional differences. Results indicated that the RF approach is a practical tool to study the connection between various influencing factors and carbon emissions in the Yangtze River Economic Belt from different perspectives. Furthermore, regional differences among the primary carbon emission influencing factors for each city were clearly observed and were related to urban population characteristics, urbanization level, industrial structures, and degree of openness to the outside world. These factors variably affected different cities, but the results indicate that regional emission reductions have achieved positive results, with overall simulation trends shifting from underestimation to overestimation of emissions.

## 1. Introduction

In order to mitigate the many negative effects caused by carbon emissions, the Chinese government signed the Paris Agreement in 2016, thereby promising to cap peak carbon emissions around 2030 and reduce carbon emissions per unit GDP to 60%–65% of 2005 levels. However, China is the most populous developing country in the world and remains in a stage of rapid economic development. Consequently, how to accurately target the main factors affecting carbon emissions in the face of huge pressure to reduce emissions has become key to achieving the goal without stifling development. Many studies in recent years have investigated economic factors, population sizes and structures, technological progress, and industrial structures via factor analysis in order to determine their relative contributions to carbon emissions.

Economic factors mainly affect carbon emissions due to economic development levels and development progress. Improvements in economic levels tend to increase consumption and production demand, which are primary factors affecting carbon emissions [1, 2]. An inverted U shape is generally observed [3] between development and emissions that is related to levels of local economic development. The economic development model thus also describes regional emission reductions. For example, long-term investment in some Chinese cities has been used to promote economic development, allowing technological progress and energy structure improvements to offset some of the effects of increased emissions [4].

Human impacts on carbon emissions are the main drivers of production and consumption activities and can be assessed from two aspects: population size and population structure. Any increase in numbers of people will lead to increased carbon emissions [5]. Concomitantly, maintaining moderate population densities can improve the efficiency of infrastructure and energy use, while knowledge sharing brought about by population agglomeration promotes energy-saving technology progress [6]. Nevertheless, excessive population densities increase regional carbon emissions due to traffic congestion and resource waste caused by excessive competition [7]. In addition to population growth, differences in population structure also impact regional carbon emissions. These components include the age structure of the population, gender ratios, family sizes, and urban population fraction [8–10].

Advancements in science and technology, improvement of energy utilization efficiency, and optimization of factory production processes have also shown promise as powerful measures to reduce carbon emissions [11]. However, much technological progress also promotes new energy-consuming industries and spurs consumer demand [12] due to lowered production costs and the use of greater resources in production [13]. These dynamics tend to offset some of the carbon emission improvements brought by technological progress. There are consequently considerable regional differences in the role of technological progress in reducing carbon emissions, and the same technology upgrades may yield better emission reduction effects in developed regions compared to less developed regions [14].

Industrial structure is also an important factor affecting regional carbon emissions. When the secondary industry is dominated by manufacturing, this sector is often the main source of carbon dioxide emission in a region [15]. Resources can be reused by integration within the industry, while developing low-emission high-value-added service industries and technology-intensive manufacturing can also help effectively reduce regional carbon emission levels [16, 17].

In addition to the aforementioned factors, changes in land use patterns [18], differences in urban structures [19, 11], and foreign trade [20, 21] are also key factors affecting carbon emissions. Interactions between these influencing factors result in variable changes in carbon emissions, and the role of each influencing factor during different time periods or in different regions also varies. Thus, in-depth analysis of changes in influencing factors is critical for achieving carbon emission reduction targets and is also the basis for proposing feasible emission reduction measures.

Several research methods are available for analyzing carbon emission influencing factors, with the most commonly used methods being path analysis [22], panel data models [23], spatial econometric models [24], the logarithmic mean Divisia index (LMDI) [25, 26], and the Kaya identity [27]. Path analysis, panel data models, and spatial econometric models are all parameterized statistical models. Prior to such analyses, certain statistical premises need to be met. For example, strong noncollinearity between factors is required to ensure the required minimum variance of parameter estimations. LMDI, the Laspeyres index method, and the Kaya identity are all factor decomposition models that require satisfaction of an identity relationship. This in turn leads to the weakening of influencing factors and may lead to heavily one-sided interpretations of results. Such model restrictions can lead to incomplete planning considerations when developing feasible emission reduction measures, thereby rendering it more difficult to give accurate guidance and recommendations.

Machine learning is a generic term for algorithms that imitate the learning processes of human beings by adopting autonomously generated computer models. Machine learning algorithms can continuously improve their operation processes based on a training data set, or it can combine multiple learning results into a more comprehensive and accurate model to achieve integrated learning and obtain optimal results. In addition, machine learning features the advantages of variable selection not being limited by data collinearity, thereby allowing the development of more stable and objective results. Random Forest (RF) classification is a widely used machine learning algorithm and features advantages of strong resistance to overfitting, good adaption to large-scale data, and variable selection that is not restricted by collinearity. RF algorithms have been previously used to investigate carbon sinks in soils and forests [28, 29]. However, little research has investigated the application of RF to influencing factors involved in national and regional carbon emissions (e.g., [30]). Furthermore, the application of RF algorithms to city-level carbon emissions has not been evaluated. Consequently, a RF algorithm was used in this study within a machine learning environment to analyze the characteristics of urban carbon emission factors in three urban agglomerations in the Yangtze River Basin in order to explore the key factors that affect urban emission reductions.

## 2 Data and methods

### 2.1 Study area

Seventy-three cities from the three major urban agglomerations of the Yangtze River Economic Belt (YREB) were used in this study including the Yangtze River Delta Urban Agglomerations, the Middle Yangtze Urban Agglomerations, and the Chengdu-Chongqing Urban Agglomerations. The Yangtze River is the third longest river in the world and is an important link connecting China’s eastern coastal areas and inland areas. The YREB comprises 11 provinces and cities including Shanghai, Jiangsu, Zhejiang, Anhui, Jiangxi, Hubei, Hunan, Chongqing, Sichuan, Yunnan, and Guizhou. The YREB area comprises about 2,052,300 square kilometers, accounting for 21.4% of China’s land mass. In addition, the total population and GDP of the region both represent over 40% of China’s totals. The shipping conditions allowed by the Yangtze River have created strong internal economic and social connections across the entire region. In addition, the broad regional area leads to significant differences in the natural, economic, and social conditions of these cities and regions. The primary carbon emission factors of the cities in the region thus mainly encompass unique spatial characteristics. The areas of the 73 cities cover the entire range of the YREB belt, from upstream to downstream. Furthermore, the 73 cities exhibit different development stages that are characterized by different carbon emission levels, industrial levels, and other social and natural characteristics. The carbon emissions in these cities are also driven by varying factors including population characteristics, secondary and/or tertiary industries, and technology, as previously described.

### 2.2 Data

The carbon emissions of each city were first calculated. A city’s carbon emissions primarily come from energy consumption, industrial production processes, and waste disposal [31, 32]. The data necessary to account for carbon emissions from industrial production processes and waste disposal are missing for some cities. Thus, to ensure a unified accounting of emissions among our dataset, energy carbon emissions were selected as a proxy for total urban carbon emissions, with reference to the accounting method described in the “2006 IPCC Guidelines for National Greenhouse Gas Inventories” document [33]. The carbon emissions of each province and city were then calculated and included the effects of thermal power generation, agriculture, industry, building industry, wholesale and retail industry, accommodation industry, transportation, warehousing, postal services, and residential consumption. Relevant economic and social indicators were selected with reference to previous studies, and carbon emission data for each province were reduced to the city level based on the proportion of city indicators in that province [34–36]. The specific indicators that were selected are shown in Table 1, with data taken from the “China Energy Statistical Yearbook” and the statistical yearbooks of various provinces and cities between 2000 and 2017.

**Table 1:** Economic and social indicators for allocating urban carbon emissions.

Industry or sector generating carbon emissions	Economic or social indicators
Industry	Industrial added value
Agriculture, forestry, animal husbandry and fishery	Primary industry added value
Thermal power generation	Electricity consumption in society
Building industry	Building construction area
Wholesale and retail, Accommodation, transportation, warehousing,	Tertiary industry added value
resident consumption	Permanent Residents

The specific calculation formula that was used was:

$$C_{x}={\sum^{n}}_{i=1}(E_{i}\times CEF_{i}\times NCV_{i}\times COF_{i})$$ (1)

where *C*_*x*_ is the total energy consumption carbon emission of the x department of the province; *n* is the energy type; *E*_*i*_ is the i^th^ type of energy consumption; *CEF*_*i*_ is the carbon emission factor per unit calorific value of the i^th^ energy type; *NCV*_*i*_ is the i^th^ energy type’s low calorific value of energy; and *COF*_*i*_ is the carbon oxidation factor of the i^th^ energy source. The necessary reference values were taken from the “General rules for comprehensive energy consumption calculation” (GB / T 2589–2008) [37] that was formulated by the Chinese government and the “2006 IPCC Guidelines for National Greenhouse Gas Inventories” [32]. For city y,

$$C_{y}={\sum^{m}}_{x=1}C_{x}\times Q_{yx}/Q_{x}$$ (2)

where C_y_ represents the total carbon emissions of city y, m represents the type of sector, Q_x_ represents the economic or social indicator of the x^th^ department of the province where the y^th^ city is located, and Q_yx_ represents the economic or social indicator for department x of city y.

**Fig 1:**
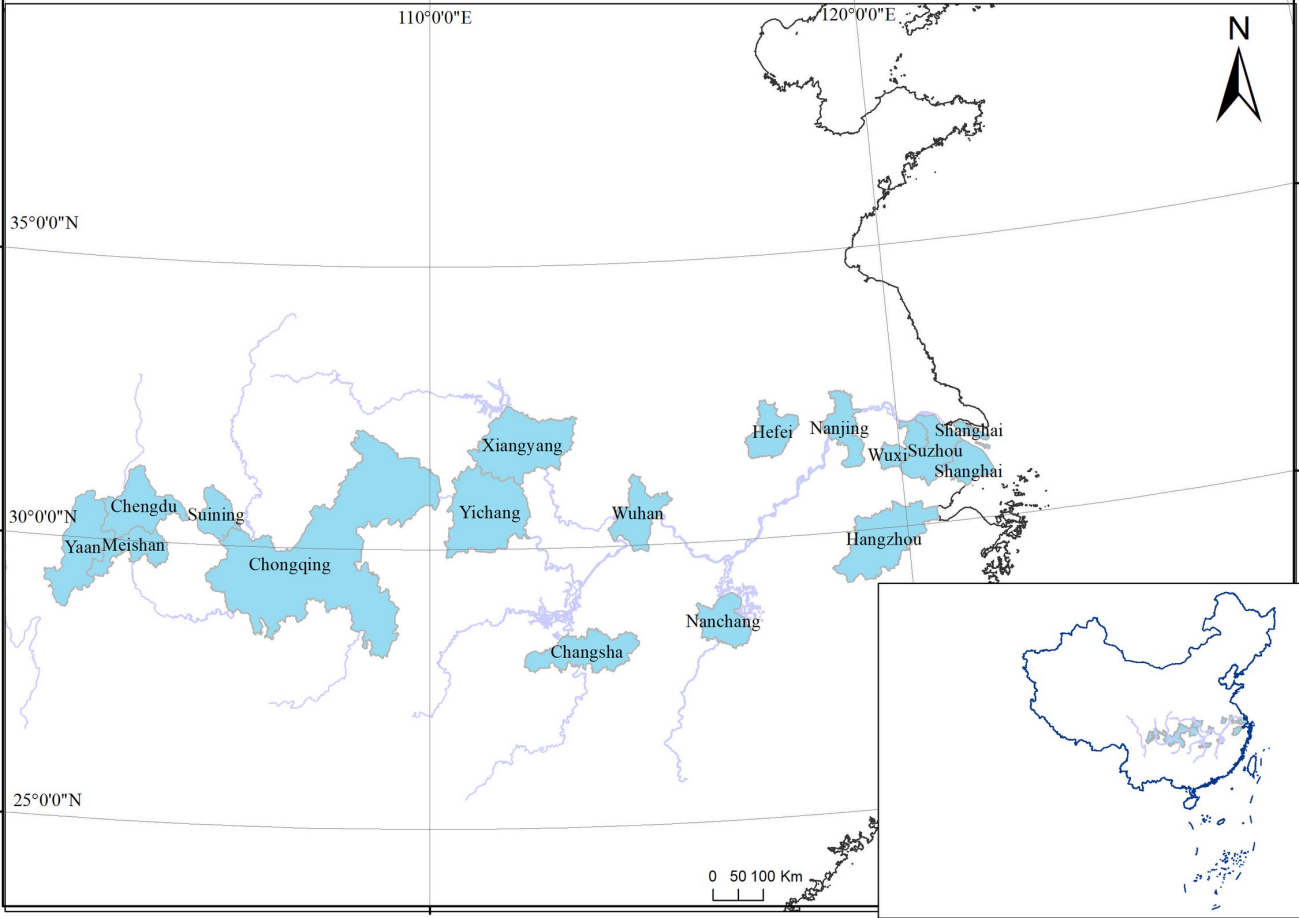
The geographic location and city names of 16 cities with data from 2000 to 2017. Areas of cities are shaded by colours while the Yangtze River is indicated by lines. Data source are retrieved from Ministry of Natural Resources of the People’s Republic of China (grant number: NO. GS(2020)4621; http://bzdt.ch.mnr.gov.cn/index.html).

Carbon dioxide emissions (CDE) data for 73 cities located in central and southeastern China (Fig 1) were included in this study from recent years in addition to data for ten influencing factors (Table 2) considered to be potentially related to CDE. Previous studies were used to guide the selection of influencing factors, taking into account data availability. Overall, the factors focus on five areas: populations (PR and PUP), industrial structures (PSI and PTI), technical levels (LP and PTAGE), household consumption (PCCEUR and PCCERR), and the degree of openness to the outside world (IUTTH and FD).

**Table 2:** Ten influencing factors and the short names used.

Influencing Factors	Short Names
Proportion of Secondary Industry	PSI
Proportion of Tertiary Industry	PTI
Labor Productivity	LP
Proportion of Technology Appropriation to Government Expenditure	PTAGE
Internet Users per Ten Thousand Households.	IUTTH
Per Capita Consumption Expenditure of Urban Residents	PCCEUR
Per Capita Consumption Expenditure of Rural Residents	PCCERR
Permanent Residents	PR
Proportion of Urban Population	PUP
Foreign Dependency	FD

Population agglomeration and increases in urban population are likely to affect regional carbon emissions. In particular, the permanent resident (PR) population and the proportion of urban population (PUP) are used to indicate both population sizes and population structures. Industrial structure is another key factor affecting carbon emissions. In this study, the proportion of secondary industry (PSI) and proportion of tertiary industry (PTI) were selected based on the proportion of added value from various industries in a city’s GDP to represent urban industrial structures. Labor productivity (LP) was used to represent the level of industrial production technology. In addition, the proportion of technology appropriation to government expenditure (PTAGE) was used to represent the government’s investment in scientific research activities that in turn represents the city’s technological level in terms of practical applications and technical reserves. Residential consumption is divided into two indicators based on differences in consumption levels and consumption concepts between urban and rural residents: the per capita consumption expenditure of urban residents (PCCEUR) and the per capita consumption expenditure of rural residents (PCCERR). The number of internet users (IUTTH) is used to proxy the convenience with which people in the region can obtain external information in order to estimate degree of openness. The foreign dependence (FD) degree indicates the degree of foreign investment in local production. Data were retrieved from the statistical yearbooks and statistical bulletins for the cities, the “China City Statistical Yearbook,” the “China Regional Economic Statistical Yearbook,” and the “Yangtze River Delta and Pearl River Delta, and Hong Kong, Macao and Taiwan Statistical Yearbooks,” with interpolation to estimate some missing values. All abovementioned data source are clarified in S1 Table.

### 2.3 The RF algorithm

RF is a recently developed supervised learning algorithm [38, 39]. Compared to other algorithms that similarly attempt statistical downscaling (canonical correlation analysis, classification and regression trees (CART), and neural networks), RF outperforms nonlinear approaches and also achieves better performance when dealing with complex data [40]. In particular, RF techniques offer the most improvement over CART methods that use a tree-like process to relate inputs to grouped outputs using layers [41]. CART methods split nonlinear relationships between inputs and outputs into several ranges so that the nature of the internal relationships can be simplified, thereby allowing them to be modelled using simple linear processes or constants [42]. Splitting progress is executed by repeatedly dividing the tree into branches until simple regression between inputs and outputs is statistically evident [43]. Compared to standard CART analysis, RF features two additional layers of randomness [44] including that a larger set of trees can be obtained using numerous bootstrapping iterations to generate a forest and, second, that each tree in an RF is fitted using a random collection of predictors.

The most relevant parameters in an RF are thus 1) the number of trees generated (NT), 2) the number of maximum predictors available for each tree (NP), and 3) the maximum number of terminal nodes each tree in the forest can have (NN). RF can be used for classification and regression, and when used for regression, RF outputs are the average of all outputs obtained from each tree. The most important advantage of RF is that it outperforms CART while reducing the possibility of overfitting [45]. The relative importance of each predictor on the prediction is estimated for each tree by calculating the increase in the mean square error caused by amending a given predictor. Detailed mathematical and statistical information for the RF algorithm is described in previous studies including Sall (1990), Breiman (2001, 2002), Ishwaran (2007), and Grömping (2009) [46–49].

## 3 Results

### 3.1 City responses to CDEs

**Fig 2:**
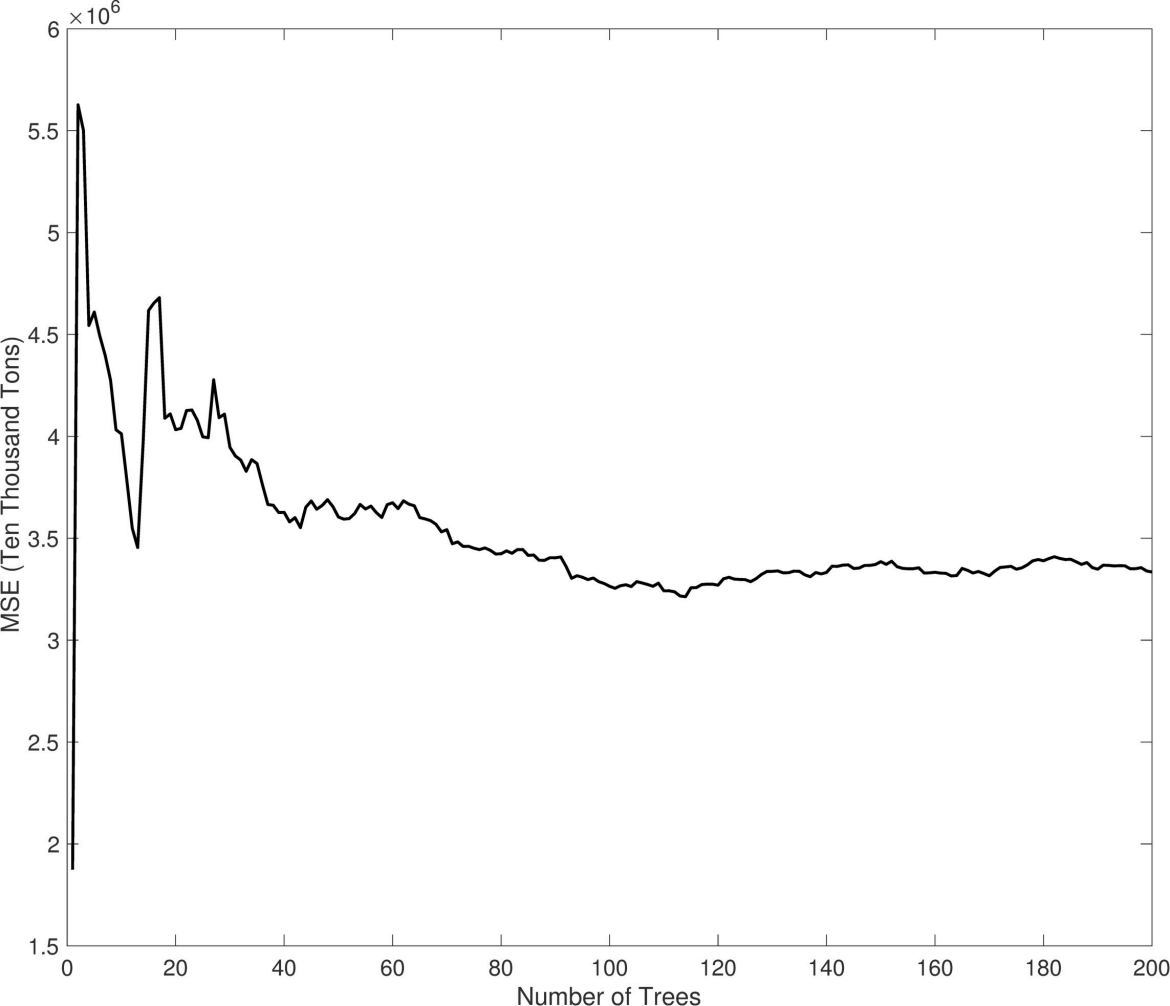
Curve of the mean squared errors resulting from RF varying with the number of decision trees (NT).

An RF was generated by applying 292 observations drawn from the 73 cities across several years to determine the variation in CDE change for each city. Specifically, the years considered were 2000 (the earliest time point with relatively complete data in the study area), 2007 (the year when China surpassed the United States to become the world’s largest carbon emitting country), 2012 (the intermediate node for the next 10 years), and 2017 (the regional energy balance sheet used to calculate carbon emissions was updated to 2017). The RF used predictors indicating time (corresponding to years) and cities to model the corresponding CDEs. Selections of NT, NP, and NN were made by looping each set of possible values to individually fit the RF, thereby determining the RF that provided optimized estimations. For each step of the loop, a leave-one-out cross validation (LOOCV) process with 10 folders was applied to test the performance of the RF. This method divides data into 10 equal portions and uses one portion as test data, with the other nine portions used as training data to independently train each classifier. The predictions from the 10 classifiers for the corresponding testing data were then combined into a vector of the same length as the original data (292 elements), and R^2^ was calculated. Full looping iterations (not shown here) revealed that an RF with NT = 200, NP = 53, and NN = 70 exhibited optimal performance based on the highest observed R^2^ value (0.842). The NT choice was further confirmed by calculating the mean square errors (MSEs) from RFs with 1< = NT < = 200 (Fig 2), revealing that the MSE series tended to be constant when approaching 100 NT, indicating that an NT = 200 choice is sufficient.

**Fig 3:**
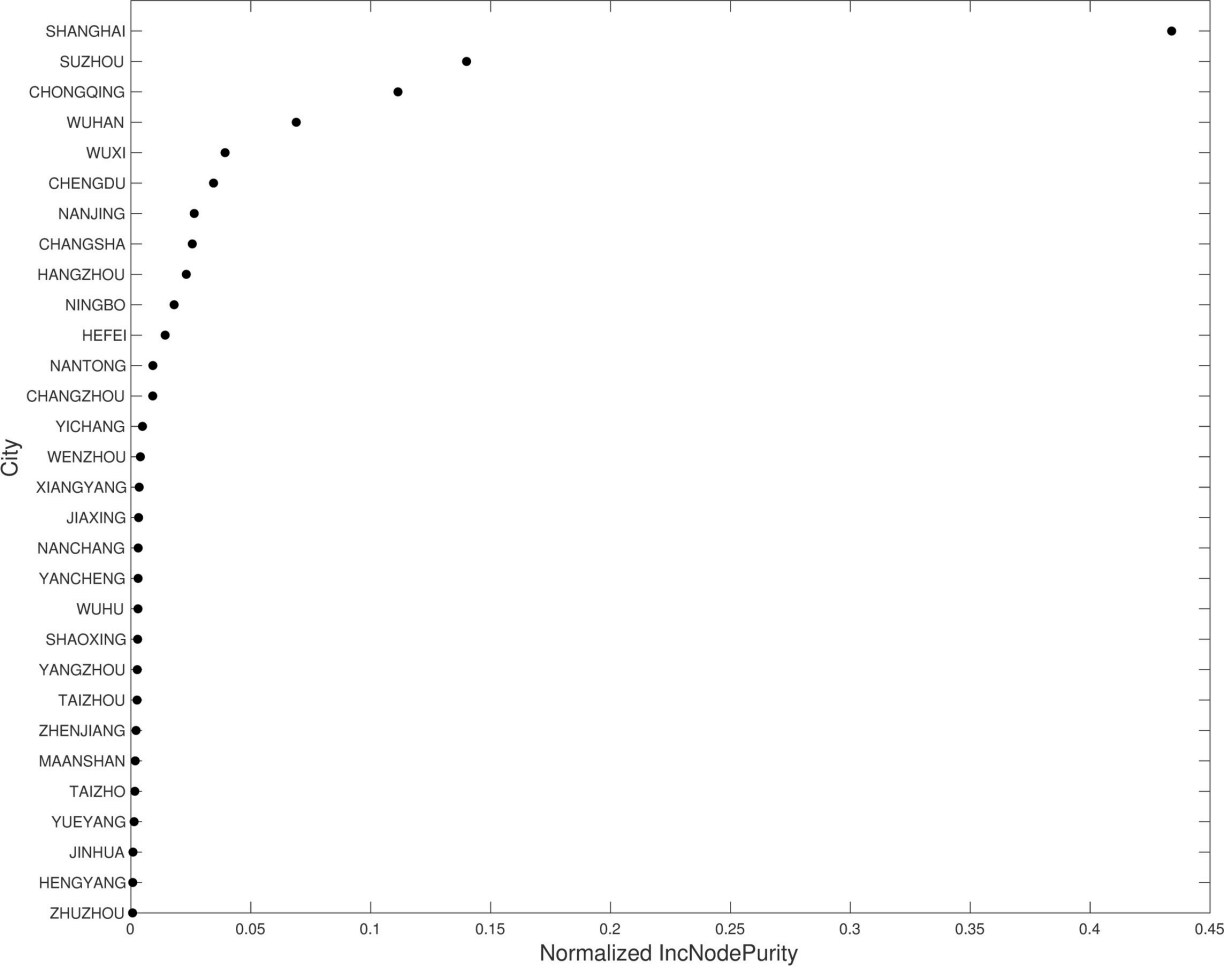
The top 30 normalized Gini impurities resulting from RF.

RF reflects the purity of the attribute division of the decision tree based on the size of the Gini coefficient. Smaller Gini coefficients indicate higher purity of the classification nodes within a decision tree. For these calculations, all nodes are divided, and the reduction of the average Gini coefficient is calculated, wherein the greater the node reduction, the greater the impact of that node on the entire forest, and thus the higher importance of the node. This effect represents the Gini impurity, and normalized Gini impurities for various cities are shown in Fig 3. Here, only the first thirty cities with the highest Gini impurities are shown. Thus, variation in economic levels, industrial structures, and population sizes among cities lead to significant spatial difference in the ranking of Gini impurities among cities. Overall, cities in the Yangtze River Delta city group that are located in the eastern coastal areas had higher values, including Shanghai, Suzhou, Wuxi, Nanjing, Hangzhou, Ningbo, and Hefei. Previous studies have shown that this effect is related to the size of the urban population and economy within a region, including strong energy demands and energy structures that are dominated by coal use [50–52]. The Gini impurity rankings for cities were generally low in the Middle Yangtze Urban Agglomeration and Chengdu-Chongqing Urban Agglomerations, with the exceptions of Chongqing, Wuhan, Chengdu, and Changsha. These results are consistent with previous studies of the spatial distribution of carbon emissions in Chinese cities. The total quantity of emissions exhibited a gradual decreasing trend when moving from eastern to western regions [53–55].

### 3.2 Representative city analysis

**Fig 4:**
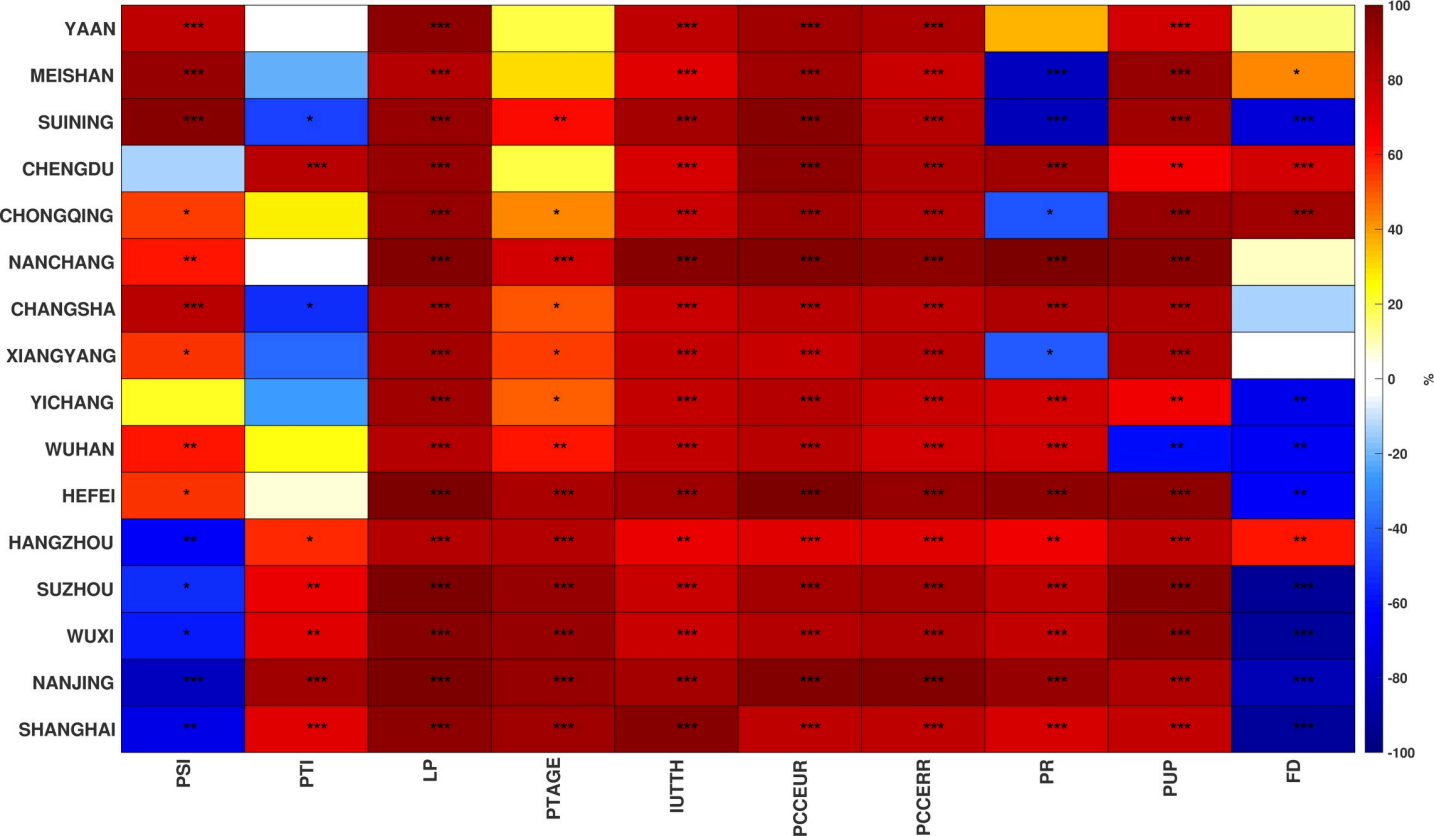
Correlation between 10 influencing factors and carbon emissions during 2000–2017 in 16 cities. Each row represents a particular city, while each column represents a particular influencing factor. Colours in each pixel represent correlation, while labels *, **, *** separately indicate statistical significance in 90%, 99%, 99.9% confidence intervals.

As shown in Section 3.1, the carbon emissions of some cities have undergone significant changes in recent years. Sixteen representative cities were subsequently selected from the YREB region by combining the geographic location of each city and its normalized Gini impurity value, as calculated in Section 3.1 (Fig 1). The correlations between 10 influential factors and carbon emission data between 2000 and 2017 are shown in Fig 4. The correlations between carbon emissions and LP, IUTTH, PCCEUR, and PCCERR were significantly positive in all 16 cities, implying that these factors may promote carbon emission increases in these cities. The correlations between carbon emissions and PR were significantly positive in most cities, but tended to be negative or insignificant in cities with population losses (Fig 4). The correlations between carbon emissions and FD were only significantly positive for a few cities, implying that the dependence of carbon emissions on industry and companies brought by foreign investment became weak during 2000–2017. The correlations between carbon emissions and PSI and PTI exhibited geographically variable characteristics. In downstream cities of YREB (e.g., Shanghai), the correlations between carbon emissions and PSI were significantly negative, while those with PTI were significantly positive. However, opposite patterns were observed in the middle stream and upstream cities of YREB. This result is generally consistent with variation in the industrial source of carbon emissions among YREB cities, while industrial carbon emissions in downstream cities are primarily contributed by tertiary industries, and those in other YREB regions are contributed by secondary industries.

**Fig 5:**
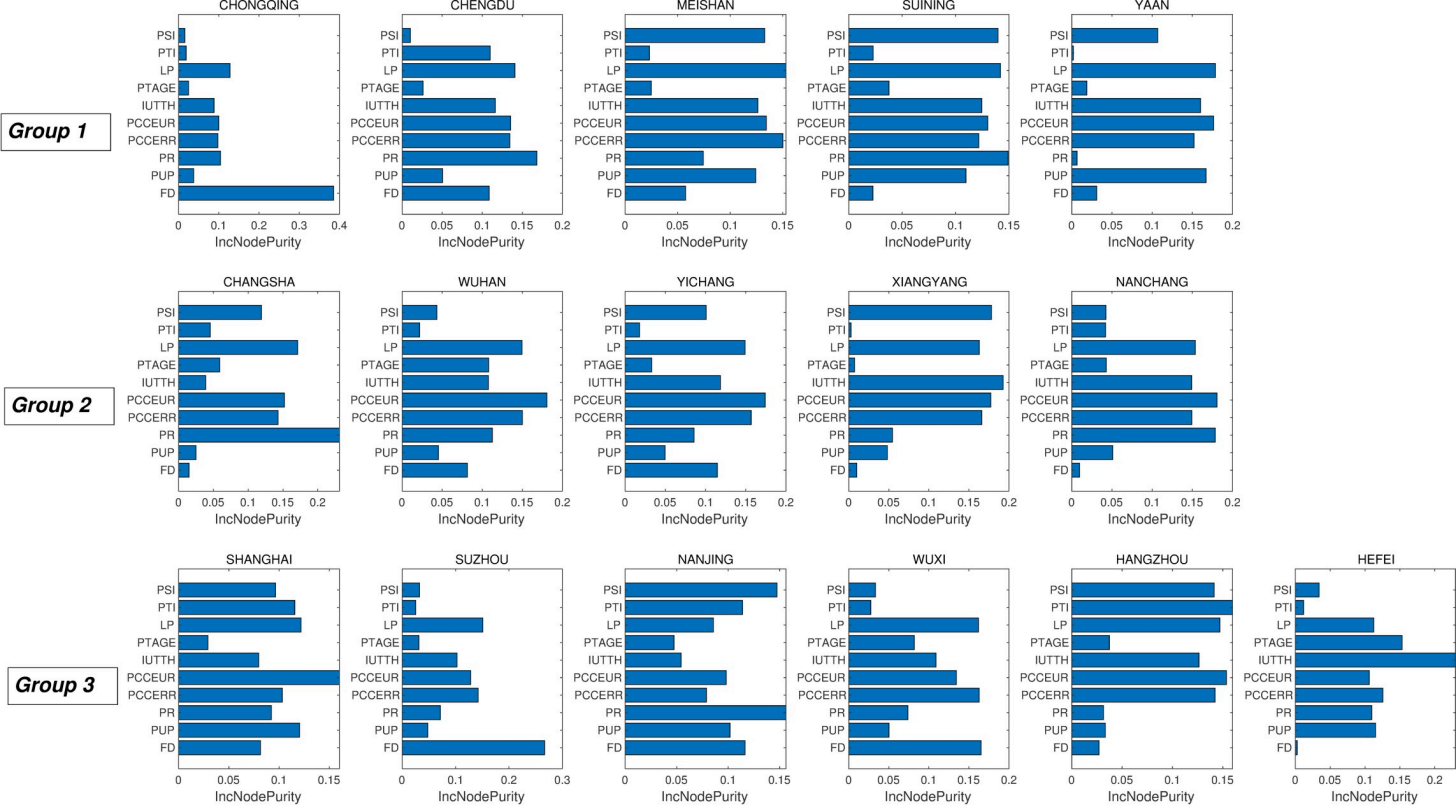
The Gini impurity of 10 impact factors for 18 cities. The plot is organized following the order of three city agglomerations introduced in Section 3.2 (Groups 1–3). Each panel represents an individual city, labelled in the title of each panel.

Correlational analysis yielded obvious connections between carbon emissions and the 10 evaluated influencing factors. Nevertheless, it is hard to directly quantify the contribution of each influencing factor to carbon emissions using only correlational analysis. The carbon emission data from 2000 to 2017 and data for the 10 influencing factors (Table 2), along with data for each city in each year, were used as observation inputs to establish an RF to fit the CDE. The RF was independently fitted to the data for each city, and the determination of relevant parameters (NT, NP, and NN) was conducted for each city following the steps described in Section 3.1. The aforementioned correlational analysis revealed that the associations between influencing factors and carbon emissions varied geographically. Consequently, the 16 selected cities were divided into three urban agglomerations to evaluate the normalized Gini impurities for each influencing factor for the 16 cities (Fig 5).

The cities in Group 1 comprised the Chengdu-Chongqing Urban Agglomerations in the upper reaches of the Yangtze River. Chongqing and Chengdu are the primary cities in the region, and investigation of the influencing factors of urban carbon emissions in the region resulted in the following observations.

The average level of urban economic development in this region is low compared with the middle and lower reaches of the Yangtze River, as shown by small PCCEUR values during 2000–2017 (S1 Fig) [2], with production processes that require improvement, leading to LP values having a higher influence on CDE. Despite the urbanization levels of other cities rapidly increasing, rural residents still account for much of the city populations in this agglomeration (with the exception of Chongqing and Chengdu), as indicated by a small PUP in cities of the region (S1 Fig). The PCCERR and PUP are consequently the main causes of local carbon emissions. In addition, production factors like population and capital are concentrated in these cities due to the polarizing effect of Chongqing and Chengdu on the economic and social resources of the surrounding areas [56]. Furthermore, PR and FD consequently have greater impacts on the carbon emissions of these cities, and each city’s influencing factors expresses unique characteristics.

The FD effect on Chongqing’s carbon emissions is much higher than for other influencing factors and is probably related to Chongqing’s policy advantages for its municipalities. Chengdu’s industrial structure is dominated by tertiary industry (S1 Fig), and this has contributed to increases in population and consumption. PTI, PR, PCCEUR, and PCCERR are the main sources of increased carbon emissions in this area primarily due to the fact that the three cities Meishan, Suining, and Yaan have large secondary industries (S1 Fig). PSI thus significantly promotes carbon emissions in this region. In addition, the region has undertaken industrial transfers from other metropolises [57], and the transfer of energy-intensive heavy industries has potentially further increased the observed LP Gini impurity.

Group 2 comprised the Middle Yangtze Urban Agglomeration, with Wuhan and Changsha being the primary cities of the region. Due to the path dependence of industrial structures (S1 Fig), LP was the most important influencing factor for each city, while suppressed effects of PSI as a carbon emission influencing factor were observed for all cities except Xiangyang. The impact of consumption on local carbon emissions increased over time, but the PCCEUR and PCCERR Gini impurities differed between cities and were related to the urbanization level of each city (S1 Fig).

Targeted analyses for each city indicated that the tertiary industry represented by commercial circulation and finance has begun to develop in Changsha. In 2017, the output value for tertiary industry accounted for 50.5% of the city’s GDP (http://tjj.hunan.gov.cn/tjfx/tjgb/szgb/zss/201804/t20180403_4986306.html). PR, PCCEUR, and PCCERR are the main causes of carbon emissions in Changsha, as indicated by relatively high Gini Impurities (Fig 5). Similarly, PR and PCCEUR also influenced Wuhan’s carbon emissions. Previous studies have confirmed that Wuhan is an important transportation hub in the region [58], and the resulting accumulation of populations and goods promotes economic development in the city, thereby generating increased carbon emissions.

The urbanization rates of Nanchang, Xiangyang, and Yichang all were in rapid development stages in 2017 (S1 Fig). However, the impact of changes in household consumption on carbon emissions was more obvious when compared with population agglomeration. The impact of PCCEUR on carbon emissions in these three cities is clearly increasing. The number of internet users in both cities is not as high as in other core regional cities (e.g., Shanghai and Chengdu), but the number of internet users in these cities is still rapidly increasing (S1 Fig), and IUTTH is thus becoming more important. Increased access to the internet creates new industries and jobs [59, 60]. However, the PSI rankings among the three cities’ influencing factors were still higher than those for PTI, suggesting that internet access has not yet promoted tertiary industry in this region. Thus, developing means to fully allow internet use in the future to promote technological progress and industrial upgrades while striving to avoid negative effects on emission reductions is a focus that these cities should specifically consider.

Group 3 comprised the Yangtze River Delta Urban Agglomerations located in the alluvial plain of the Yangtze River estuary and is one of the areas with the most active urbanization development in China. The long developmental history of the region has led to a well-established industrial system and good divisions of labor in the area [61, 62]. The major influencing factors on carbon emissions within the cities in this area exhibited significant differences. Shanghai is the core regional city, and PCCEUR and PUP were the most important influencing factors of its emissions. Additionally, the impact of PTI on Shanghai’s carbon emissions is also distinct and is likely resultant from the strong tertiary industry in the city, including the financial industry and wholesale and retail industries [63]. However, Shanghai is an important high-end manufacturing center for electronic information products, automobile manufacturing, petrochemicals, and other industries of scale in China [64]. Thus, the impact of PSI on Shanghai’s carbon emissions remains still significant. Similar scenarios were observed in Nanjing and Hangzhou, although differences in the dominant industries between these cities leads to variation in the rankings of some influencing factors. The financial industry in Nanjing has rapidly developed in recent years, with the financial industry’s GDP accounting for 11.6% of the city’s total in 2017 [65]. FD thus impacts Nanjing’s carbon emissions to a greater extent. By contrast, Hangzhou’s information technology in addition to cultural and creative industries is more developed. With the rapid popularization of the internet in mainland China and Hangzhou’s advantageous location near Shanghai, these value-added industries accounted for 25.5% and 24.1% of value-add in 2017 (http://www.hangzhou.gov.cn/art/2017/5/9/art_1256295_8335913.html). IUTTH thus ranks higher among influencing factors of emissions in Hangzhou.

Both Suzhou and Wuxi are industrial gathering centers in the region, and they are favored by foreign investment sources due to their convenient transportation and production cost advantages. Some studies have suggested that investment in China is concentrated in the manufacturing sector [66]. However, PSI values do not clearly reflect this supposition. The number of internet users in Hefei significantly increased during 2000–2017 (S1 Fig), making it the fastest adopter of online access in the region. Hefei is a national science and technology innovation focal city and is also a member city of the World Science and Technology City Alliance and harbors many national laboratories and scientific engineering laboratories. These observations together explain the greater impact of IUTTH and PTAGE on the city’s carbon emissions.

Overall, regional differences in influencing factors on carbon emissions were generally clear in this study and can be attributed to differences in urbanization levels, industrial structures, and the economic development levels of each city. In cities with high urbanization levels, population and consumption are the primary causes of regional carbon emissions, consistent with results from previous studies [67, 68]. In some small and medium-sized cities evaluated here, the industrial structure is still dominated by secondary industry, although consumption also contributes to carbon emissions. It is thus more urgent to improve their industrial structures and increase labor productivity. The impact of PTAGE did not meet expectations as an indicator for measuring technical reserves, although a positive effect of technological progress on emission reduction has been previously shown [69, 70]. The reason for this discrepancy may be that development based on scientific and technological achievements takes time, and this connection may become more apparent in future studies. IUTTH exhibited the strongest influence in two types of cities: those in the process of rapid internet popularization, and those with a high proportion of information industry businesses within their industrial structures. However, the role of the internet in optimizing the industrial structures of the former cities is not yet apparent, and the Gini impurity for PSI is higher than that for PTI. FD is generally a high priority target for reducing carbon emissions among cities in the Yangtze River Delta Urban Agglomerations. This is especially evident for industrial cities and justifies evaluating the impact of international industrial transfer on China’s carbon emissions [71, 72].

### 3.3 RF Cross-validation of carbon emissions in 16 cities

**Fig 6:**
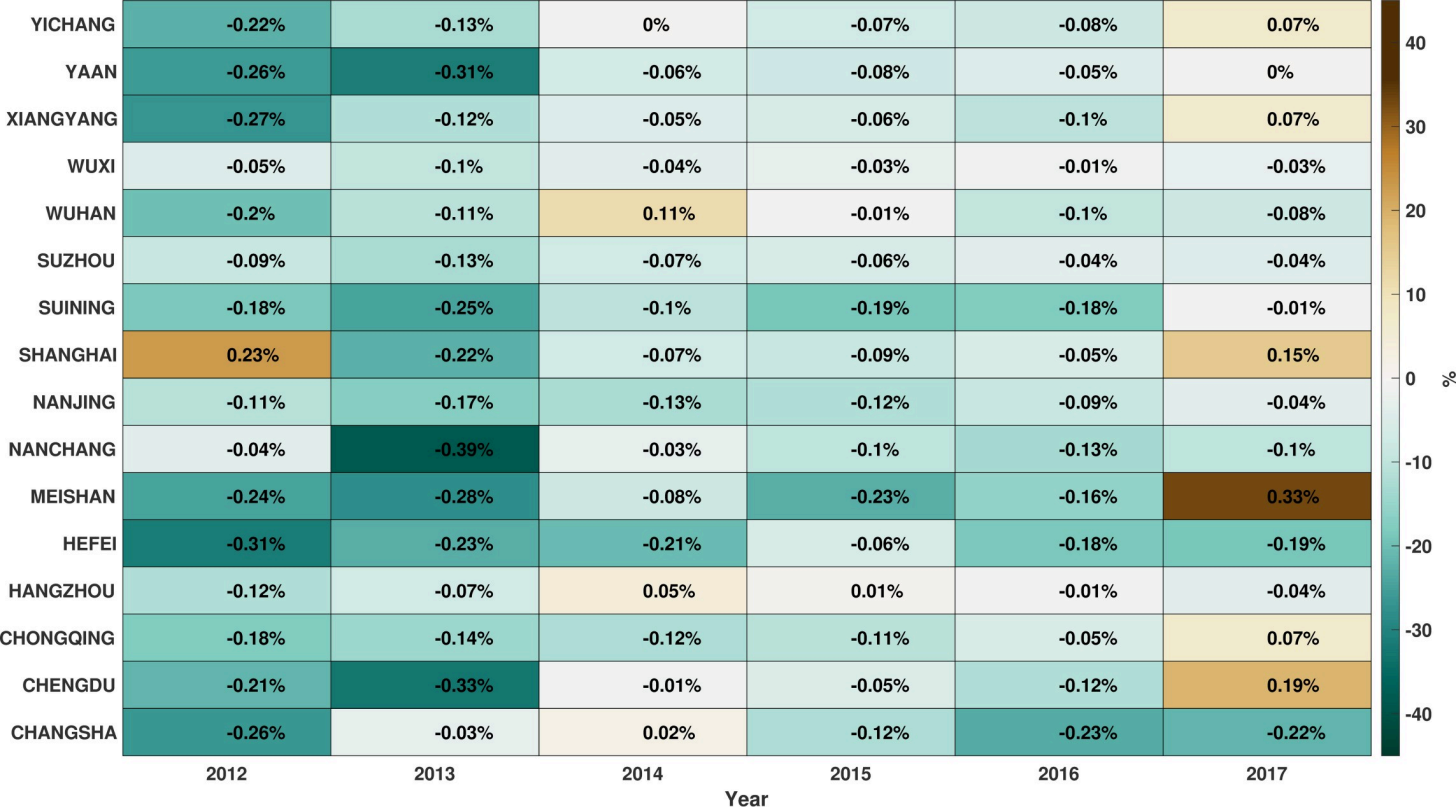
The difference (error) rate between predicted and real CDE in each city during 2012 to 2017. Each row represents a particular city, while each column represents a particular year.

RF was then used to cross-validate the carbon emissions of the 16 cities and evaluate the potential for predictability in emission rates. Specifically, the CDE of 16 cities was predicted during 2012–2017. To predict the CDE for a year, all data prior to that year were used to train the RF for each city. For example, to predict the CDE for 2014, data from 2000 to 2013 were used as the RF training set. Thus, this method allowed evaluation of how RF performance changes with increased input data. After predicting the CDE for 16 cities during 2012–2017, the difference (error) between predicted and real CDE values was calculated (Fig 6). Cross-validation results for most cities changed from underestimations to overestimations, indicating that a decoupling of urban economic development and carbon emissions in the study area has begun, consistent with results from other studies [73, 74].

The mean absolute difference of the cross-validation results was calculated by temporally averaging the absolute difference between predicted and real CDE values for each city (Table 3). Among the 16 cities evaluated, 15 exhibited an error rate of under 20%, and the average accuracy for cross-validation was 87.45%. Comparison of verification accuracy against the characteristics of each city’s carbon emission factors indicated a relationship to the primary influencing factors for emissions. For example, the mean absolute deviations for Wuxi, Hangzhou, Chongqing, and Suzhou were all within 10%, while PR and PUP were not the main emission-influencing factors in these cities. The influence of populations on carbon emissions assessed here is similar to that observed in other studies [75, 76]. The mean absolute deviations in the Middle Yangtze Urban Agglomeration fell between 11.1% and 13.8%, and an analysis of influencing factors in the region revealed that the impact of PSI is declining, while the impact of PCCEUR is increasing (Section 3.2). This is consistent with cities in the region undergoing industrial structural transformation and population agglomeration [77]. Cities in the Chengdu-Chongqing Urban Agglomeration (except for Chongqing) exhibited mean absolute deviations between 12.5% and 21.9% that were the largest mean absolute deviation values among the three urban agglomerations evaluated here. In addition to PR and PUP, IUTTH, PSI, LP, and PCCERR were also major influencing factors of emissions in this region. Thus, cities in this region are undergoing changes in both production and consumption along with urbanization and industrial transfer from coastal areas. These dynamics lead to inevitable sharp changes in carbon emissions. Improved energy use efficiency and promoting green and environmentally friendly consumption methods in the future should thus be a focus for promoting local emission reduction in this area.

**Table 3:** Mean absolute difference of 16 cities’ CDE obtained by RF cross–validation. The mean absolute difference for each city is calculated by temporally averaging all differences between predicted and real CDE (shown in Fig 5) for the corresponding city.

Rank	City	Mean Absolute Difference	Rank	City	Mean Absolute Difference
1	Wuxi	4. 82%	9	Yichang	12. 63%
2	Hangzhou	5. 93%	10	Nanjing	12. 83%
3	Chongqing	7. 24%	11	Nanchang	13. 88%
4	Suzhou	8. 24%	12	Shanghai	15. 11%
5	Xinyang	11. 16%	13	Chengdu	15. 51%
6	Changsha	11. 20%	14	Suining	17. 13%
7	Wuhan	11. 60%	15	Hefei	18. 95%
8	Yaan	12. 51%	16	Meishan	21. 93%

## 4 Conclusions and discussion

In this study, three different RF approaches were used to provide an overview of the relationships between carbon emissions and several influencing factors in the YREB. The first RF was applied to predict carbon emissions in 73 cities during 2000 to 2017 and quantify changes in carbon emissions. The second RF then used 10 influencing factors as observations to model carbon emissions in 16 selected cities based on the Gini impurity ranking and differences in geographic locations and city sizes. The results were then compared with outputs from correlational analysis. Subsequently, the third RF was used to cross-validate and predict carbon emissions in each city during 2012 to 2017 using data for the 10 influencing factors.

The primary conclusions are as follows:

1. The Gini impurity rankings for YREB cities and carbon emissions decrease when moving from eastern to western regions. However, industrial cities and regional central cities in each city cluster rank higher than cluster averages.
2. Clear differences were observed for the main influencing factors of cities. The urbanization levels of large cities are high, and their industrial structures have stabilized, resulting in PCCEUR being the main cause of their carbon emissions. Consumption in smaller cities also contributes to carbon emissions, but PSI and LP are their main emission-influencing factors. The impacts of IUTTH on carbon emissions are only significant in cities with large information industries or cities currently in the process of internet popularization. FD also exhibits obvious regional differences. Specifically, cities of the Yangtze River Delta Urban Agglomerations have generally higher influences of FD on emissions than in cities of the other two regions.
3. Cross-validation results indicate that the carbon emissions of most cities in the region have shifted in time from overestimations to underestimations. The average accuracy of this verification is 87.45%. In addition, the size of the verification error was related to a city’s main carbon emission factors. PR and PUP, respectively, reflect the size and structure of the urban population and the carbon emissions of cities. Cities with a high degree of impact from these factors like Shanghai and Nanchang are more difficult to predict. Furthermore, the influence of PR and PUP is relatively low in regions with high prediction accuracy like Chongqing and Wuxi. The cross-validation results for cities with lower rankings like Wuxi and Chongqing were also more accurate, consistent with previous studies of the influence of populations on urban carbon emissions. In addition, PSI- and LP-led industrial cities like Suining and Xiangyang exhibited higher prediction errors along with IUTTH- and PCCERR-influenced cities in industrial transformation phases like Hefei and Chengdu.

There are several implications based on these results. Emplacing emission reduction measures according to local conditions should be considered in the future based on the actual characteristics of each city. For example, when formulating emission reduction plans, large cities should consider rational control of urban population sizes and guide residents to consume in more environmentally friendly ways. Small and medium-sized cities, or cities that are in the process of industrial transformation, should prioritize improving energy use efficiency, eliminating inefficient production capacity, and actively adopting advanced management experience and production processes in conjunction with internet popularization and regional industrial transfer.

Considering the relationships between influencing factors, LP and PSI do not always exhibit strong influences at the same time. In particular, the influence of PSI has declined in some cities, while LP still plays a major role in influencing carbon emissions. Thus, a lag in the improvement of production technology is present when compared with reductions of industrial scale, and the influence of the former is stronger. International investment estimates in China have suggested that these investments are mostly concentrated in industry, and this was expected to expand the impact of PSI on carbon emissions. However, the three cities with the highest FD influence also exhibited weakened PSI influence, while the ranking of LP was higher. This suggests that international investment has a greater impact on regional carbon emissions due to production efficiency rather than by directly changing local industrial structures.

RF approaches do not require data collinearity and can be completely self-learned computationally. It thus exhibits obvious advantages for measuring urban carbon emissions factors and simulating urban carbon emissions. However, independent variable data for earlier periods in urban sites are not available, and the regional energy balance table used to calculate city carbon emissions after 2018 has not been published. It was thus not possible to further expand the dataset to make the model more complete. Furthermore, the selection of influencing factors was also not sufficiently comprehensive. The mechanisms of action for specific influencing factors should thus be explored in future studies to improve predictions.

Overall, this study confirms that RF modeling has strong applicability in analyzing urban-level carbon emission influencing factors, and the continual exploration of these models is a worthy research target. In addition, these results provide a reference for different cities for formulating emission reduction plans. Furthermore, cross-validation results confirm that China’s emission reduction efforts have already achieved some positive results. The research thus provides new ideas, a research framework, clarification of relevant carbon emission factors, and other useful information for formulating emission reduction plans for various countries or cities.

## Supporting information

S1 Fig Carbon emissions and 10 influencing factors in 16 selected cities during 2000–2017. Raw data are normalized into interval (0, 1) to regulate the unit of presented data.
(EPS)

S1 Table Name and source of data used in presented study. (DOCX)
