# Supplementary material for: Random forest analysis of factors affecting urban carbon emissions in cities within the Yangtze River Economic Belt
Source: PLoS One. 2021 Jun 4;16(6):e0252337. doi: 10.1371/journal.pone.0252337 (PMC8177642; doi:10.1371/journal.pone.0252337)
Supplement: S1 Table — (DOCX) [file pone.0252337.s002.docx]

*S1 Table. Name and source of data used in presented study.*

| *Data Name* | *Source* | *Data Name* | *Source* |
| --- | --- | --- | --- |
| *Carbon Emissions* | *China Energy Statistical Yearbook（2000-2017）* | *PCCEUR* | *China City Statistical Yearbook（2000-2017）* |
| *GDP* | *Statistical Yearbook of Shanghai, Chongqing, Zhejiang, Jiangsu, Anhui, Hubei, Hunan（2000-2017）* | *PCCERR* | *China City Statistical Yearbook（2000-2017）* |
| *PSI* | *China Statistical Yearbook for Regional Economy（2000-2014），Statistical Yearbook of Shanghai, Chongqing, Zhejiang, Jiangsu, Anhui, Hubei, Hunan, Jiangxi, Sichuan（2000-2017），Statistical Yearbook of corresponding cities（2000-2017）* | *IUTTH* | *China City Statistical Yearbook（2000-2017）* |
| *PUP* | *China City Statistical Yearbook（2000-2017）* | *LP* | *China Statistical Yearbook for Regional Economy（2000-2014），China City Statistical Yearbook（2000-2017）* |
| *PR* | *China Statistical Yearbook for Regional Economy（2000-2014），Statistical Yearbook of Shanghai, Chongqing, Zhejiang, Jiangsu, Anhui, Hubei, Hunan, Jiangxi, Sichuan（2015-2017）* | *FD* | *China City Statistical Yearbook（2003-2017），Statistical Yearbook of corresponding cities（2000-2002）* |
| *PTI* | *China Statistical Yearbook for Regional Economy（2000-2014），Statistical Yearbook of Shanghai, Chongqing, Jiangsu, Anhui, Hubei, Hunan, Jiangxi, Sichuan（2015-2017）* | *IUTTH* | *China City Statistical Yearbook（2001-2011），China Statistical Yearbook for Regional Economy（2012-2014），Statistical Yearbook of Corresponding cities（2000-2017），Yangtze River Delta & Pearl River Delta and Hong Kong&Macao SAR & Tai Wan（2000-2008）* |
